# Pre-symptomatic diagnosis and treatment of filovirus diseases

**DOI:** 10.3389/fmicb.2015.00108

**Published:** 2015-02-20

**Authors:** Amy C. Shurtleff, Chris A. Whitehouse, Michael D. Ward, Lisa H. Cazares, Sina Bavari

**Affiliations:** Molecular and Translational Sciences Division, United States Army Medical Research Institute of Infectious DiseasesFort Detrick, MD, USA

**Keywords:** Ebola, Marburg, diagnostics, therapeutics, zoonosis

## Abstract

Filoviruses are virulent human pathogens which cause severe illness with high case fatality rates and for which there are no available FDA-approved vaccines or therapeutics. Diagnostic tools including antibody- and molecular-based assays, mass spectrometry, and next-generation sequencing are continually under development. Assays using the polymerase chain reaction (PCR) have become the mainstay for the detection of filoviruses in outbreak settings. In many cases, real-time reverse transcriptase-PCR allows for the detection of filoviruses to be carried out with minimal manipulation and equipment and can provide results in less than 2 h. In cases of novel, highly diverse filoviruses, random-primed pyrosequencing approaches have proved useful. Ideally, diagnostic tests would allow for diagnosis of filovirus infection as early as possible after infection, either before symptoms begin, in the event of a known exposure or epidemiologic outbreak, or post-symptomatically. If tests could provide an early definitive diagnosis, then this information may be used to inform the choice of possible therapeutics. Several exciting new candidate therapeutics have been described recently; molecules that have therapeutic activity when administered to animal models of infection several days post-exposure, once signs of disease have begun. The latest data for candidate nucleoside analogs, small interfering RNA (siRNA) molecules, phosphorodiamidate (PMO) molecules, as well as antibody and blood-product therapeutics and therapeutic vaccines are discussed. For filovirus researchers and government agencies interested in making treatments available for a nation’s defense as well as its general public, having the right diagnostic tools to identify filovirus infections, as well as a panel of available therapeutics for treatment when needed, is a high priority. Additional research in both areas is required for ultimate success, but significant progress is being made to reach these goals.

## INTRODUCTION

Ebola and Marburg hemorrhagic fevers are caused by viruses within the family *Filoviridae; Zaire ebolavirus* (EBOV), *Sudan ebolavirus* (SUDV), *Reston ebolavirus* (RESTV), *Taï Forest ebolavirus* (TAFV), *Bundibugyo ebolavirus* (BDBV), and *Marburg marburgvirus* (MARV). These viruses are important pathogens which cause severe disease in humans and non-human primates. Currently there are no United States Food and Drug Administration-approved vaccines or therapeutics for treatment of infection with filoviruses, but due to the ongoing EBOV outbreak in West Africa in 2014, testing of new therapeutics under emergency use conditions is planned. Diagnostic tests available either in the field at the sites of the outbreak and/or in sophisticated laboratories are in use to ascertain results from samples taken from suspected cases demonstrating classical symptoms. The tests are useful, but have limitations as to the sensitivity and limits of detection for identifying a positive case as early as possible in infection. Eventually, the field of filovirus diagnostics and treatment will be positioned to consider the results from a pre-symptomatic diagnostic test to inform the selection of an appropriate therapeutic for administration of the best treatment option for a patient; however, therapeutics vetted through classical clinical research are largely unavailable, and pre-symptomatic tests are not fully developed. This review aims to describe the upcoming available therapeutics under consideration for treatment of filovirus infections, and the diagnostic tests under development to detect the infections as early as possible.

## DIAGNOSIS OF FILOVIRAL DISEASES

Rapid and accurate diagnosis of filoviral diseases is key to preventing the spread of the disease during a natural outbreak or intentional release. A range of diagnostic methods are available for the detection and identification of filoviruses. These include virus isolation, enzyme-linked immunosorbent assays (ELISAs) to detect antigen or antibodies, reverse transcriptase-polymerase chain reaction (RT-PCR) and electron microscopy, all of which have played major roles in the diagnosis of filovirus infections and have been summarized elsewhere (Kuhn, 2008; Hartman et al., 2010; Wang et al., 2011; Koehler et al., 2014). Thus, this review of the diagnosis of filoviral disease will focus on current methods used for field diagnostics during Ebola or Marburg virus outbreaks, the use of next-generation sequencing as a diagnostic tool to discover new filoviruses, and efforts to develop pre-symptomatic diagnostics for filovirus infections.

### FIELD DIAGNOSTICS

Since the first recognized occurrence of Marburg hemorrhagic disease in Germany and Yugoslavia in 1967, and the subsequent isolation of *Marburg marburgvirus* the following year (Siegert et al., 1968), sporadic outbreaks of Marburg and Ebola hemorrhagic fever have been reported from several countries in Central Africa, and most recently, the largest outbreak to date currently unfolding in West Africa (Centers for Disease Control and Prevention, 2014a,b; Meltzer et al., 2014). The largest known MARV outbreak occurred in northeastern Angola in 2004–2005, with over 250 laboratory confirmed cases identified and a case fatality rate of 90%. Another large outbreak of over 150 cases occurred in the Democratic Republic of Congo in 1998–2000, which also had a high case fatality rate of 83%. Prior to the ongoing EBOV outbreak, the largest known EBOV outbreak had occurred in Uganda in 2000–2001 and resulted in a total of 425 cases with a case fatality rate of 53%. Since February 2014, the largest Ebola disease epidemic has been occurring in West African countries of Guinea, Liberia, Sierra Leone, and Nigeria with over 8,000 deaths reported, a growing tally frequently reported with the caveat that the actual number could be much higher due to underreporting (Centers for Disease Control and Prevention, 2014a; Meltzer et al., 2014).

The differential diagnosis of filovirus infections can be challenging due to the generalized clinical signs and symptoms seen in patients early in the course of infection. Filoviral disease can be mistaken for other infectious diseases that are common in many areas of sub-Saharan Africa including malaria, shigellosis, typhoid fever, leptospirosis, yellow fever, typhus, Lassa fever, and fulminant viral hepatitis (Hartman et al., 2010). As such, early and accurate diagnosis is essential to prevent the spread of the disease. Because of this need for immediate diagnosis of filovirus disease, beginning in the early 2000s, there was an increased emphasis on bringing laboratory diagnostics to the field. Leroy et al. (2000) reported the first field evaluation of an RT-PCR assay for EBOV. Two years later, Drosten et al. (2002) developed a one-step, real-time RT-PCR method for EBOV utilizing the DNA-intercalating dye SYBR green I and the primer set originally developed by Sanchez et al. (1999) for traditional (i.e., gel based) RT-PCR. The introduction of real-time RT-PCR was a significant advancement in the area of field diagnostics as it is not only a highly sensitive method, but it also allows for the detection of filoviruses with minimal manipulation and equipment, and can provide results within about 3 h of the samples arriving in the laboratory (Drosten et al., 2002). One of the main problems in designing effective molecular diagnostics for RNA viruses is the considerable genetic variability of these viruses. To address the challenge of diversity of filoviral genomes in RT-PCR-based assays, Zhai et al. (2007) developed a consensus RT-PCR method using a cocktail of specific primers designed to the L gene and validated this method with all filovirus strains known at the time. The assay had the added value of producing a sufficiently long amplicon (640-nt product) to be sequenced for automated phylogenetic analysis, allowing accurate placement of newly identified filovirus samples relative to existing species or strains. The same year, Panning et al. (2007) reported on the development of the first industry-standard diagnostic real-time RT-PCR assay kit that was validated using all known filoviruses in the strain collections of all European BSL-4 laboratories. Likewise, 4 years later, Ogawa et al. (2011) developed a universal filovirus RT-PCR assay using primers specific for the viral nucleoprotein gene; however, this assay was gel-based, as opposed to real-time.

While RT-PCR assays are highly sensitive, fast, and accurate and have become the first choice diagnostic technique for detection of filoviruses, they require the use of high-precision thermal cyclers or real-time PCR machines. In contrast, RT loop-mediated isothermal amplification (LAMP) is a simple and rapid technique that allows for reverse transcription and DNA amplification in one step under isothermal conditions (60–65°C), thereby obviating the need for a thermal cycler (Notomi et al., 2000). Moreover, LAMP of positive samples can be evaluated in real-time by monitoring the turbidities of the reaction mixtures or by naked-eye judgment with the addition of a fluorescent dye to the reaction mixture (Tomita et al., 2008). RT-LAMP assays have been developed for EBOV and MARV (Kurosaki et al., 2007, 2010), and they have the potential to significantly improve field diagnosis for filoviruses. It should be noted that while RT-LAMP assays hold great promise for filovirus field diagnostics, they have been shown to be slightly less sensitive compared to equivalent TaqMan RT-PCR assays (Kurosaki et al., 2007, 2010).

### USE OF NEXT-GENERATION SEQUENCING FOR DIAGNOSTICS

Several consensus RT-PCR assays have proven successful in detecting all filoviruses known at the time (Panning et al., 2007; Zhai et al., 2007; Ogawa et al., 2011). However, given the genetic diversity and rapid mutation rates of RNA viruses, it is highly likely that these assays will be unable to detect novel filovirus species and lineages that emerge in the future. In fact, this diagnostic gap was highlighted when, in 2007, clinical specimens containing a newly-discovered Ebola virus species, BDBV, from Uganda tested negative with highly sensitive real-time RT-PCR assays specific for all filoviruses known at that time (Towner et al., 2008). This virus was ultimately characterized by whole genome sequencing using the pyrosequencing approach developed by 454 Life Sciences Inc., (now Roche) and subsequently, the genome sequence was used to develop a BDBV-specific real-time RT-PCR assay (Towner et al., 2008).

Recent advances in nucleic acid sequencing technologies (referred to as ‘next-generation’ sequencing [NGS]) have revolutionized the field of viral diagnostics. These technologies provide high speeds and number throughputs that can produce an enormous volume of DNA sequence data. NGS involves the extraction of the total nucleic acids from a sample, conversion from RNA to cDNA (if starting with an RNA sample), shearing them to a uniform size, and adding specific adapters to the ends of the DNA. Sequencing takes place from these adapter sequences in a massively paralleled manner using any one of several next-generation sequencing platforms currently available (i.e., Illumina, PacBio, Ion Torrent, and others). The main advantage in clinical diagnostics is that there is no need to design specific primers to pre-amplify target sequences. The disadvantage is that these methods also allow for the sequencing of all host DNA in the sample thus necessitating the need for powerful bioinformatics to find the ‘needle in the haystack.’ Applications of NGS in virology have included whole genome sequencing, discovery of new viruses by using metagenomics approaches, analysis of viral genome variability (i.e., quasispecies), identification of viral communities (i.e., viromes) in the environment and in the human body or animal models, and identification of antiviral drug resistance mutations (Barzon et al., 2011; Capobianchi et al., 2013; Lecuit and Eloit, 2014). In addition, the availability of having large numbers of sequenced genomes afforded by NGS allows for more robust phylogenetic analyses. For example, investigators have made great strides in studying the global ecology and phylogeography of influenza viruses by analyzing several thousands of sequences (Lam et al., 2013; Nelson et al., 2014). Thus, it is likely that in the near future unbiased NGS will become the primary tool, not only for routine diagnosis of infectious diseases, but to identify novel pathogens and to elucidate their ecology and epidemiology.

The utility of NGS in the discovery of new species/variants of Ebola viruses has already become evident. First, as mentioned above, the BDBV was originally characterized by 454 pyrosequencing (Towner et al., 2008). In addition, Negredo et al. (2011) described the first Ebolavirus-like filovirus from Europe with the help of next-generation sequencing. This virus, called Lloviu virus, was identified from dead insectivorous bats in Spain and represents the first filovirus identified in Europe. As more researchers examine more potential hosts using high-throughput NGS, it is possible that more novel filoviruses may be discovered. Throughout the 2014 Ebola outbreak in Guinea and Sierra Leone, investigators have used NGS methods to understand how EBOV moved across African nations during the months of the outbreak, to describe the virus sequence’s differences and similarities to previous outbreak variants and to describe its emergence from the natural reservoir (Gire et al., 2014). In a study compiling 81 sequences of Sierra Leonean and Guinean origin, phylogenetic comparison of these sequences to those from previous outbreaks indicated that this 2014 West African EBOV likely originated from central Africa within the last decade (Gire et al., 2014). The 81 sequences demonstrate similarity, indicating the progenitor virus emerged once from its reservoir into the human population, and sustained virus transmission has been between humans, rather than multiple reintroductions from the reservoir (Gire et al., 2014). Looking forward, the information garnered about the virus sequence from NGS studies will enable researchers to evaluate the accuracy of diagnostic tests currently in use, and the design and efficacy of antiviral strategies, such as small molecule drugs and vaccines under development.

### PROSPECTS FOR PRE-SYMPTOMATIC DIAGNOSIS

A diagnostic test that could identify infected patients before the onset of symptoms, but after exposure to an infectious agent, would be an indispensable tool for guiding the individual’s potential treatment options and/or mitigating potential epidemic spread of the disease. Much of the work in this area has been focused on identifying specific gene expression signatures in the peripheral blood of patients infected with various viruses, bacteria, or fungi (Pankla et al., 2009; Tang et al., 2009; Berry et al., 2010; Zaas et al., 2010; Mejias et al., 2013, 2014). However, these studies were not truly pre-symptomatic as they focused on patients with active infections at the peak of their symptoms. In some studies, blood-based gene expression data have been able to distinguish patients with viral infections from those with bacterial infections as well as healthy controls (Ramilo et al., 2007; Zaas et al., 2009). Much of the recent work in this field has focused on the detection of influenza and other respiratory viruses and identifying virus-specific gene expression profiles at some early time points after virus exposure. In experimental viral challenge studies conducted on healthy human volunteers, investigators were able to diagnose influenza virus infection using an acute respiratory viral bio-signature 45 h after challenge, while the median time to peak symptoms was 80 h post-infection (Zaas et al., 2009). In another more recent study, an influenza-specific gene expression pattern, which is comprised of an array of almost 50 host response genes known to be responsive to viral infections, was detectable in a blood RNA sample. The signature was detectable as early as 29 h post-exposure for H3N2 and 38 h for H1N1 infections, and achieved a sensitivity of 89% without false positives at 53 h for H3N2 and 60 h for H1N1 influenza (Woods et al., 2013). The timing and severity of symptoms varied greatly depending on the individual patient and type of influenza virus used; however, the average time to first symptom onset for H3N2 was 49 and 61 h for H1N1. The sensitivity was increased to 100% as time passed, but by the time such solid sensitivity was achieved, the peak symptomatic period was already in full swing. The magnitude of virus load in these patient blood samples was not reported in these studies therefore, it is unclear if virus should have been detectable in the blood or other samples by RT-PCR at the same time as these gene signatures were detectable. Therefore, this type of host genomic analysis has the potential to identify viral infection before symptoms emerge when early intervention with antiviral medication could have a profound impact on symptoms, transmission, and disease outcome.

**FIGURE 1:**
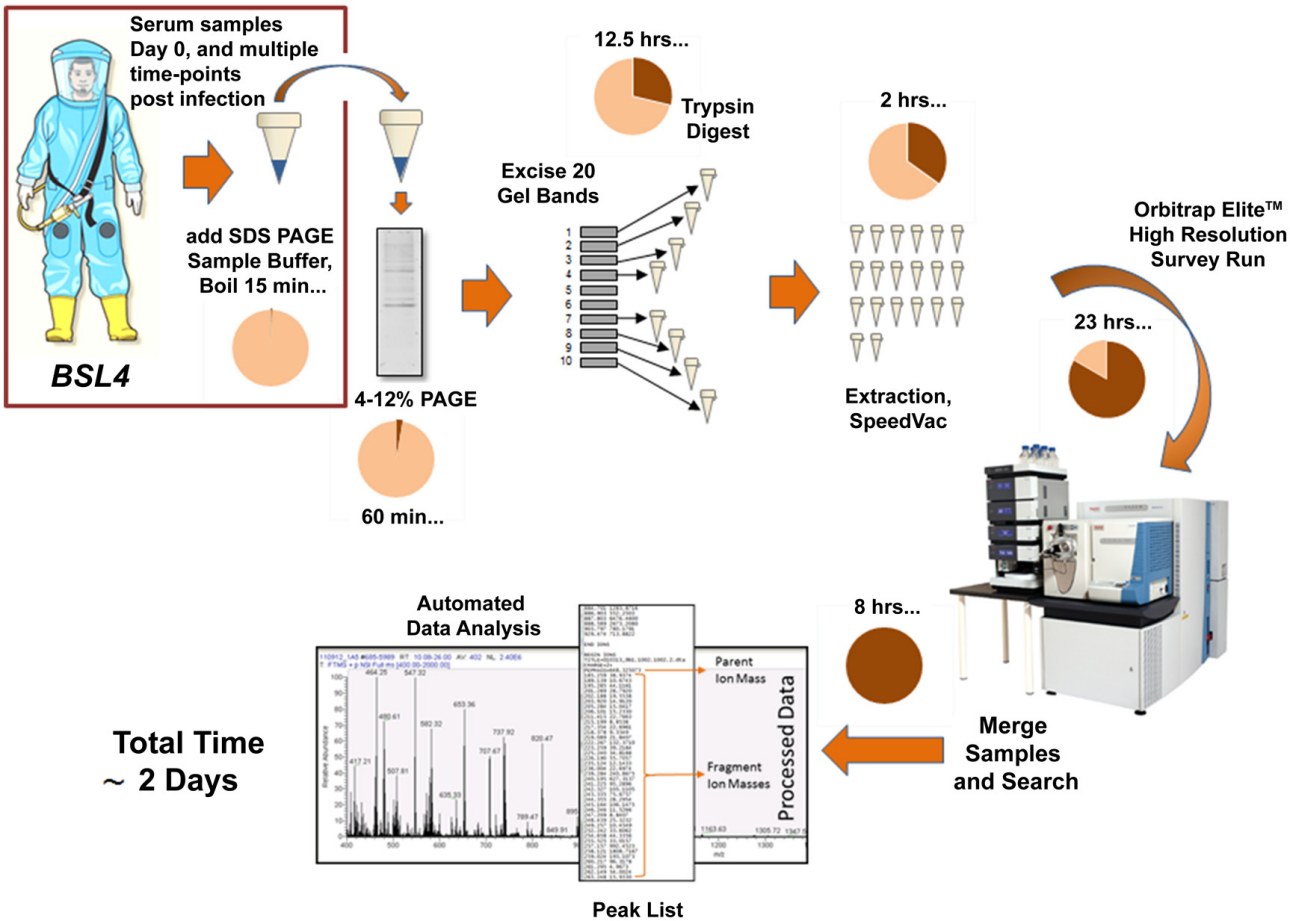
**Current workflow for the detection of filoviral proteins and host response proteins in serum samples from Ebola virus-infected NHPs.** The use of SDS PAGE sample buffer and boiling provides inactivation of the virus allowing downstream processing to take place in a BSL-2 environment.

Proteomic methodologies and state of the art mass spectrometers provide a complementary approach to genomics technologies by examining the protein content of complex samples, yet these approaches are not realistic for use in resource-limited areas. Many studies have used such techniques and instrumentation for the detection and discovery of biomarkers in serum samples for a variety of disease states, including cancer, diabetes, and neurodegenerative disorders, to enable early and accurate diagnosis (Zeng et al., 2010; Isabel Padrao et al., 2012; Jia et al., 2012; Fan et al., 2013; Jimenez and Verheul, 2014; Liang et al., 2014; Taylor et al., 2014). A recent study using in-depth proteomic analysis of serum samples from patients infected with HIV, demonstrated for the first time that acute phase proteins are induced systematically prior to the first detection of viremia and also before any detectable increase in plasma cytokine levels (Kramer et al., 2010). Unfortunately, little work on pre-symptomatic detection of filoviruses has been performed to date. However, efforts are underway to screen filovirus infected non-human primate serum (NHP), to determine which viral proteins are first shed into the blood and which host response proteins may be indicative of the disease or are immune correlatives of infection. To examine host response to filovirus infection, pre-infection serum samples are compared against post-infection samples to determine fold change of serum and viral proteins that can be detected each day post-infection (see **Figure 1**). Differentially expressed host proteins and viral proteins shed into the blood over the course of early filovirus infection may be identified. Preliminary results indicate that viral proteins can be detected early in infection, with the first detectable peptides originating from the GP1 molecule. In addition to the membrane-associated GP present on the viral membrane, the Ebola viruses also encode for a secreted non-structural glycoprotein (sGP; Volchkov et al., 1995; Sanchez et al., 1996; Falzarano et al., 2006). Previous studies have demonstrated that sGP induces a host antibody response that focuses on epitopes it shares with GP1 and GP2, thereby allowing it to bind and compete for anti-GP1 and GP2 antibodies, providing an immunomodulatory or decoy function (Basler, 2013). Similarly, the Lassa virus soluble glycoprotein 1 has been detected in infected human serum via Western blot during active infection, before the detection of virion-associated proteins such as nucleoprotein (Branco et al., 2010). Therefore, it appears that there may be a window in the early stages of infection where soluble forms of viral glycoproteins can be detected, and before whole virions are present in the bloodstream of the host. The exploitation of this biological phenomenon may lead to pre-symptomatic diagnostic assays as well as potential therapeutics.

In preliminary analyses of infected NHP serum by mass spectrometry, we have also observed many host proteins which display increased expression as filovirus infection progresses, such as complement components and acute phase proteins. While it remains to be seen whether any of these changes are specific to filovirus infection, the characterization of the host response may provide potential targets for therapy. Ultimately, we envision a biomarker panel encompassing both host response proteins and viral protein detection for the development of a more accurate early diagnosis platform. Although many researchers and physicians feel that liquid chromatography mass spectrometry (LC-MS/MS) platforms are too complex and insufficiently robust for clinical applications, mass spectrometry workflows are already widely used for clinical chemistry and toxicology analytes, and blood-based drug measurements (Takino, 2013). With sufficient sensitivity, accuracy, and throughput, it is feasible for MS-based platforms to transition to a clinical application for filovirus diagnostics if they can achieve sufficient sensitivity and specificity (Lehmann et al., 2013; Wu and French, 2013). However, it will be critical to optimize methods and instrumentation efficiencies to reduce the assay time to 1 day, if possible.

### ANIMAL MODELS AND FILOVIRUS DETECTION

Experimental viral challenge studies in non-human primates have provided the most informative data regarding the natural history of filoviral infection and host responses to infection. In particular, these studies have addressed questions such as what is the earliest time after infection that viremia, or other biomarkers of disease, can be detected. The answer to that question will certainly be affected by the animal model, the type of detection assay used, its sensitivity and its lower limit of detection. The dose and species of challenge virus affect the progression of disease, however (**Table 1**). For example, SUDV given intraperitoneally at a dose of 1000 guinea-pig infectious units caused slower disease progression than EBOV when given at the same route and dose level in experimentally-infected rhesus and cynomolgus macaques (Ellis et al., 1978; Fisher-Hoch et al., 1992), but SUDV infection by the aerosol route was shown to produce a similar disease course in three different species of non-human primates when administered at either 50 or 500 plaque forming unit (PFU) doses (Zumbrun et al., 2012). Similarly, Reston virus, which is not considered to be virulent in humans due to discoveries of human seroconversions to Reston virus but no past observation of hemorrhagic disease in these individuals, is clearly less pathogenic in monkeys than EBOV and SUDV in African green monkeys and cynomolgus macaques (Fisher-Hoch et al., 1992; Jahrling et al., 1996). Little is known, however, about the pathogenesis of *Taï Forest ebolavirus* or BDBV in non-human primates. MARV infection in cynomolgus macaques is a well-characterized model for Marburg hemorrhagic fever disease in humans. After an incubation period of 4 or 5 days, monkeys showed febrile illness, anorexia, and petechial skin rash when infected with the Angola or Ci67 variants of MARV (Alves et al., 2010; Hakami and Alves, 2010; Hensley et al., 2011). Animals infected by aerosol with 100 PFU of MARV variant Angola became febrile 4–7 days post-exposture, and viremia, as measured by plaque assay with a lower limit of detection likely at 100 PFU/mL plasma, coincided with the onset of fever at days 4–6 (Alves et al., 2010). By contrast, in another study, animals infected intramuscularly with MARV Ci67 variant did not show signs of clinical illness until day 5, but 33% of animals displayed low plasma viremia probably at or just above the level of detection (around 10–100 PFU/mL, detected by plaque assay) on day 3 post-infection and 100% of animals by day 4 (Hensley et al., 2011). In another filovirus model study, EBOV viremia was detected by modified plaque assay in serum and urine at a level of about 100 PFU/mL as early as 24 h after subcutaneous infection of rhesus macaques with a high infectious dose (10^5^ guinea-pig LD50s) of EBOV (Fisher-Hoch et al., 1985). In rhesus and cynomolgus macaques infected with 1000 PFU of EBOV-Zaire, however, viremia was first detected 3 days after infection (Geisbert et al., 2002), while viremia occurred 4–5 days after infection in baboons exposed to 20–50 newborn mouse LD_50_ of EBOV (Ryabchikova et al., 1999). In studies where qRT-PCR was used to measure viral genome equivalents in serum, values began to increase on days 3–4 post-exposure (Twenhafel et al., 2013). Clearly, when determining the earliest detection of viremia, or biomarkers of viral infection, in experimental animal models, the virus species/strain, species of NHP, inoculum level, and route of infection must all be taken into consideration (**Table 1**).

**Table 1:** Characteristics of selected NHP models of filoviral diseases.

Virus	Viral species/strain	Animal species	Infecting dose	Route of infection	Earliest onset of symptoms	Earliest detection of infection	Method of detection	Reference
Marburg	Angola	Cynomolgus	99–705 PFU	Aerosol	Days 4–7 PE (fever)	Days 4–6 PE (viremia)	Plaque assay	Alves et al. (2010)
Marburg	Ci67	Cynomolgus	1000 PFU	IM	Day 5 PI (rash)	Day 3 PI (viremia)	Plaque assay	Hensley et al. (2011)
Ebola	Zaire (E718)	Rhesus	10^5^ guinea pig LD_50_	IP	Days 4–5 (prostration, dehydration, and weight loss)	Day 4 (neutrophilia)	CBC w/differential	Fisher-Hoch et al. (1983)
Ebola	Zaire (E718)	Rhesus	10^5^ guinea pig LD_50_	IP	Day 4 (fever)	Day 1 (low-level [10^2^ PFU] viremia and neutrophilia)	CBC and plaque assay	Fisher-Hoch et al. (1985)
Ebola	Zaire	Baboons	20–50 newborn mouse LD_50_	Subcutaneous	NR	Day 4 (viremia); Day 5 (viremia)	Newborn mice assay; plaque assay	Ryabchikova et al. (1999)
Ebola	Zaire	Rhesus	9.8 × 10^2^–2.7 × 10^5^ PFU	Aerosol	Day 5 PE (fever)	Day 4 (viremia)	qRT-PCR	Twenhafel et al. (2013)
Ebola	Sudan	African green monkey	500 PFU	Aerosol	Day 3 (fever)	Day 4 (viremia)	qRT-PCR	Zumbrun et al. (2012)
Ebola	Sudan	Cynomolgus	500 PFU	Aerosol	Day 5 (fever)	Day 4 (viremia)	qRT-PCR	Zumbrun et al. (2012)
Ebola	Sudan	Rhesus	500 PFU	Aerosol	Day 3 (fever)	Day 5 (viremia)	qRT-PCR	Zumbrun et al. (2012)

*IM, intramuscular; IP, intraperitoneal; NR, not reported.*

Other than viremia, are there other markers of infection that can be detected in the peripheral blood? In one study of aerosolized EBOV in rhesus macaques, lymphocyte counts initially increased, but began to decline sharply on day 2 post-exposure (Twenhafel et al., 2013). Similarly, mean platelet counts peaked on day 3 and steadily declined thereafter. In contrast, neutrophil counts initially declined, but began to increase on day 3 post-exposure (Twenhafel et al., 2013). In fact, lymphopenia, thrombocytopenia, and neutrophilia appear to be prominent features of EBOV infections in non-human primates (Fisher-Hoch et al., 1985; Jaax et al., 1996; Twenhafel et al., 2013). Unfortunately, most of the changes seen with these hematological markers either do not all uniformly occur within the presymptomatic period and/or they are not specific to filovirus infections.

A recent study was published in which the investigators examined the levels of 55 different biomarkers, such as cytokines, chemokines, acute phase proteins, coagulation and fibrinolysis factors, from 187 serum samples from 86 SUDV-infected Ebola hemorrhagic fever patients from the 2000–2001 outbreak in the Gulu district of Uganda (McElroy et al., 2014). While this study was not designed to determine pre-symptomatic biomarkers of SUDV infection, the authors showed that elevation of levels of certain cytokines and chemokines, such as IL-1α, IL-1RA, IL-6, MCP-1, MCSF, and MIP-1α, correlated with fatal outcomes, which was consistent with other studies on SUDV-infected patients (Hutchinson and Rollin, 2007). Death and hemorrhage were also associated with elevated thrombomodulin and ferritin levels (McElroy et al., 2014). In Zaire EBOV-infected rhesus macaques, Ebihara et al. (2011) found the proinflammatory cytokines IL-1β, IL-6, and MIP-1α to be elevated, but these elevated levels were not seen until day 4 post-infection. Similarly, in a cynomolgus macaque model of Marburg hemorrhagic fever, increased levels of IFN-α, IL-6, MIP-1α, MIP-1β, MCP-1, and eotaxin, but not until late stages of the disease (days 6–8 post-infection; Hensley et al., 2011). In that study, no detectable increases in levels of any cytokines or chemokines were observed in the early or mid-stages of disease. We will continue to compare these findings with our own observations of host response proteins detected using the MS-based platform, but more work will need to be performed before pre-symptomatic diagnosis of filoviruses becomes a reality.

## FUTURE AND CURRENT TREATMENTS FOR FILOVIRUS INFECTIONS

Even before the 2014 Ebola outbreak in West Africa became the largest outbreak on record (Baize et al., 2014; Briand et al., 2014), there was a push in therapeutics discovery research to find effective treatments that could be initiated therapeutically, i.e., well-after exposure to an infectious agent, and preferably after symptoms manifest. For years, a common experimental design approach in basic research was to administer a candidate therapeutic to an animal model of filovirus infection either before virus challenge, concurrently with challenge, or very soon thereafter, and certainly before any signs of disease were apparent. Among other reasons, this prophylactic approach was probably taken to lower the bar for the candidate therapeutic and give it the best possible chance of working against a virulent virus that replicates quickly. Once such a mark of success was observed, then the dose regimen and timing could be altered for experimental designs that could prove a drug candidate to be therapeutic in nature.

### BLOOD-PRODUCTS OR ANTIBODY-BASED TREATMENTS

Whole-blood transfusions from convalescent survivors is an acceptable medical treatment in western Africa, and historical clinical data from the Kikwit 1995 outbreak support this practice as somewhat effective, even though the past trials of these methods do not conclusively demonstrate efficacy. Eight female healthcare workers who had contracted the disease received transfusions, and 7 out of 8 survived, which is a significantly lower case fatality rate than the general rate for the 1995 Kikwit outbreak, which was over 80% (Mupapa et al., 1999). One important observation made about this study of eight patients is the superior supportive care they received compared to the general population during this epidemic, which included hydration, electrolytes, glucose, anti-infectives, and food supplementation (Mupapa et al., 1999; Sadek et al., 1999). Significant challenges to use of whole-blood transfusions or other human-origin blood-products such as hyperimmune serum in treatment of disease are the need for HIV- and hepatitis B and C virus free products and the performance of tests to ensure such product safety for the recipients (Sadek et al., 1999).

In response to the infection of two American healthcare volunteers in Liberia during the 2014 Ebola outbreak, an experimental therapeutic comprised of three monoclonal antibodies against Ebola glycoprotein, called ZMapp, (Mapp Biopharmaceutical Inc., San Diego, CA, USA) was made available (Goodman, 2014). After treatment of these two patients with ZMapp, these two patients did improve, but by treatment in the absence of a properly controlled clinical trial setting, it is not possible to claim that the treatment is responsible for their survival. In the days that followed the administration of this monoclonal antibody therapeutic, which had never before been tested in humans, there occurred a significant amount of debate between a panel of twelve experts assembled by the World Health Organization about the ethics of testing drugs in only early stages of clinical or preclinical development, and whether the potential benefits of offering such drugs to Ebola infected patients would outweigh the safety risks in administering a compound heretofore untested in humans (Goodman, 2014; Sayburn, 2014). A handful of studies available in the peer-reviewed literature describe ZMapp predecessor antibodies, MB-003 and ZMab, and their efficacy in monkey studies (Olinger et al., 2012; Pettitt et al., 2013; Qiu et al., 2013; Qiu and Kobinger, 2014). The MB-003 monoclonal antibody cocktail protected 43% of rhesus macaques, which had developed both a fever in response to challenge with 1067 PFU EBOV and measurable viral genomic material in the blood by positive RT-PCR test. ZMab has been shown in cynomolgus macaques to provide 100% protection to *n* = 4 animals when given at 24 h post-infection, but only 50% protection was observed when it was given at 48 h post-infection (Qiu et al., 2012). While these studies demonstrate antiviral efficacy in *in vivo* studies, they had small experimental group sizes, and the studies were not designed to assess the safety of these Mab cocktails; however, no overt side effects were noted in the experimental monkeys. The ZMapp therapeutic is comprised of optimal antibodies as a monoclonal antibody cocktail, and is 100% effective in NHP challenge studies when 6 of 6 Rhesus macaques were given the ZMapp1 therapeutic up to 5 days post-infection (Qiu et al., 2014). Analysis of published sequences has shown that epitopes targeted by ZMapp have not changed from the experimental EBOV-Kikwit variant tested in NHP studies to the Guinean variant circulating in the current outbreak, and this neutralizing activity was confirmed through antibody binding studies and plaque reduction neutralization tests (Qiu et al., 2014).

The human efficacy and safety of the ZMapp product is still uncertain, since news reports of the current outbreak have reported that one Spanish priest who may have received ZMapp has died of Ebola infection, and two or three African healthcare workers have also received ZMapp; with only one death in that group of treated patients. There are reports of antiviral drugs like lamivudine and brincidofovir being tried in treatment of EBOV-infected patients, with no solid data on whether they are truly efficacious (Bishop, 2015). There is also serious consideration of a variety of other therapeutics, such as favipiravir (Fuji Film/Toyama Chemical), TKM-Ebola (Tekmira), AVI-7537 (Sarepta), and BCX-4430 (Biocryst), for use in human phase 1 clinical trials in Africa. Some of these compounds are either in some stage of advanced development, or maybe have never been in humans before, so the feasibility of phase 1 studies is still under investigation. In response to the outbreak, various government funding agencies have called for offerors such as MAPP Biopharmaceutical Inc., and other entities to propose novel Ebola or filovirus treatments to be the basis of novel drug discovery and development programs; therefore, studies with ZMapp and other interesting therapeutics are underway, with data forthcoming.

### ANTISENSE AND SMALL-MOLECULE THERAPEUTICS

Another drug candidate supported by funding from the US Department of Defense, TKM-Ebola, is under rapid development by Tekmira Pharmaceuticals of British Columbia. TKM-Ebola is a combination of three siRNA molecules designed to block production of VP24, VP35 and the RNA-dependent RNA-polymerase in EBOV- infected cells. These siRNAs have been chemically modified to make them somewhat resistant to endogenous endonucleases, and formulated in self-assembling stable nucleic acid-lipid particles, which are thought to facilitate delivery to the liver (Geisbert et al., 2010b). This therapeutic protected 2 out of 3 monkeys, when tested at 2 mg/kg administered by intravenous bolus doses, at 30 min post-virus challenge, and again at days 1, 3, and 5, for a total of four doses (Geisbert et al., 2010b). Another similar experiment using four EBOV infected monkeys was designed to administer the combination siRNA therapeutic at 30 min, and each of days 1 through 6 post-infection, and that regimen resulted in 100% survival. All of the animals in this second experiment appeared to have clinical disease signs consistent with severe EBOV infection, but viremia appeared to be well-controlled, never reaching higher than 1000 PFU/mL of plasma (Geisbert et al., 2010b). Since February 2012 TKM-Ebola has been under testing in a randomized, single-blind, placebo-controlled phase 1 clinical trial to evaluate single and multiple ascending doses for human safety. While TKM-Ebola was put on full clinical hold in July 2014 by the US FDA to elucidate the mechanism of cytokine release observed at higher doses, and for modification to the multiple ascending dose study protocol to ensure safety of healthy volunteer subjects, as of August 7, 2014 the FDA has modified the restriction to a partial clinical hold, so that the compound can be tested in the EBOV outbreak where it might possibly offer some protective benefit in sick patients.

Tekmira has also demonstrated the protective efficacy of their lipid-encapsulated siRNA technology when it is designed to combat infection of guinea pigs with three different MARV variants (Ursic-Bedoya et al., 2014). Two siRNAs designed to target the nucleoprotein of MARV at two different locations were the most effective against the Angola variant of MARV, with one of them showing complete protection. One siRNA against the NP gene for the RNA-dependent RNA-polymerase was 40% protective against MARV-Angola, but the siRNAs tested for activity against VP24 and VP40 conferred no protection to the guinea pigs (Ursic-Bedoya et al., 2014). Additionally, the MARV siRNAs against NP showed some protective efficacy against the Ci67 and Ravn variants of MARV when given as a cocktail (Ursic-Bedoya et al., 2014).

Two recent studies with an antiviral molecule under advanced development for treatment of influenza infections, favipiravir (T-705), have shown some efficacy in mouse models of Ebolavirus infection (Oestereich et al., 2014; Smither et al., 2014). T-705 is a viral RNA polymerase inhibitor with broad activity against various families of viruses including arenaviruses, bunyaviruses, alphaviruses, orthomyxoviruses, and paramyxoviruses (Furuta et al., 2009, 2013; Buys et al., 2011; Gowen et al., 2013; Safronetz et al., 2013; Scharton et al., 2014). Most of these broad spectrum data were collected in *in vitro* studies or small animal model studies, but T-705 is a mature compound which has been tested in several completed US FDA phase 1 and 2 clinical trials, and is currently under evaluation in phase 3 trials in Japan. In an initial oral administration study, the model of infection of A129 IFN alpha/beta receptor knockout mice by the aerosol route with 1 TCID_50_ of EBOV was uniformly lethal by day 8 (*n* = 6). Mice receiving twice daily oral doses of 150 mg/kg T-705, starting 1 h post-challenge and continuing for 14 consecutive days, completely survived the infection and recovered to day 30 post-infection (*n* = 6; Smither et al., 2014). Soon afterward, another study was published demonstrating T-705’s therapeutic efficacy when given as late as 6 days post-infection (Oestereich et al., 2014). In this study, C57/BL6 IFN alpha/beta receptor knockout mice were inoculated intranasally with 1000 PFU of EBOV and then T-705 was given twice daily by oral gavage on days 6 to 13 at a dose of 300 mg/(kg × d; *n* = 5). All five mice survived the infection when the dosing began at day 6, but when the same dosing regimen was tried starting at day 8, the mice did not survive (*n* = 5). Importantly, viremia had reached titers of around 10^4^ FFU/mL by day 6 when the treatment began, and once treatment started, the virus load began to drop significantly compared to controls. In addition, other clinical signs of infection, such as weight loss, temperature elevation, serum aspartate aminotransferase (AST) and alanine aminotransferase (ALT) elevations had presented by day 6, and began to return to normal in the T-705 treated groups. While the mouse model is not as stringent as the NHP model, this study is the first to demonstrate success of a candidate therapeutic in an animal model in which the animals were showing clear clinical, biochemical and virological signs of disease, providing a signal for diagnosis and informing when to start treatment (Oestereich et al., 2014).

Another viral polymerase inhibitor under investigation for therapeutic efficacy is BCX4430 (Warren et al., 2014). This novel nucleoside analog, with favorable pharmacokinetic properties, has shown *in vitro* antiviral activity at the micromolar concentration range against an impressive list of negative-stranded RNA viruses (Warren et al., 2014). In a mouse model of Ravn virus (RAVV) infection, which is a filovirus in the genus Marburgvirus, mice given twice daily doses of 150 mg/kg BCX4430 as late as 96 h post-infection were 100% protected from lethal RAVV infection. BCX4430 was also protective to EBOV infected mice when given twice daily by intramuscular or oral routes, but starting 4 h prior to viral inoculation and continuing through day 8. No therapeutic data for EBOV infection in these mice were reported. BCX4430 was further tested in two guinea pig models of MARV infection: one in which 1000 PFU MARV-Musoke was administered intraperitoneally, and one in which 700 PFU MARV-Angola was administered by aerosol challenge. In these models, 100% protection was seen in the Musoke model when BCX4430 was given twice daily at 50 mg/kg intramuscularly as late as 72 h post-virus inoculation, and 75% protection was seen in the aerosol Angola model with the same dose regimen. It is unlikely that disease signs were observed prior to treatment initiation as late as 72 or 96 h post-virus challenge in either of the mouse or guinea pig model experiments described here, and details to this effect were not reported (Warren et al., 2014).

BCX4430 was also tested in cynomolgus macaques for efficacy against wild-type MARV infection. NHP were solidly protected from lethal infection when BCX4430 was given at 24 or 48 h post-infection, and delivered as a twice-daily intramuscular dose at 15 mg/kg. This study is particularly important because overall it is probably the first time a small molecule therapeutic has shown efficacy in a NHP model of filovirus infection, and moreover, when that molecule was administered as late as 48 h post-infection. Even though the NHP in this study had not progressed to overt illness prior to initiation of treatment, this aspect of testing will be evaluated as this candidate therapeutic advances through preclinical and clinical development under the FDA’s Animal Rule (21 CFR 314.600).

One more set of promising therapeutics against MARV and EBOV infections comes in the form of a new class of positively charged phosphorodiamidate morpholino oligomer (PMO)s called PMO*plus*. The stable PMO molecules resemble single-stranded DNA, and form duplexes with specific target RNA sequences, thereby interfering with mRNA translation, and affecting viral RNA transcription and translation. The PMO*plus* chemistry, designed such that the oligomer contains a limited number of positively charged linkages within its structure, is responsible for enhanced efficacy and improved binding kinetics (Swenson et al., 2009; Warren et al., 2010). Comprehensive reviews are available about PMO chemistries and applications (Stein, 2008; Iversen et al., 2012), as well as their efficacies in cell culture or small models of filovirus infection (Enterlein et al., 2006; Warfield et al., 2006; Spurgers et al., 2008; Swenson et al., 2009). Warren et al. (2010) demonstrated that post-exposure protection by a combination PMO*plus* antisense therapeutic, AVI-6003, comprised of two PMO*plus* antisense molecules (AVI-7287 and AVI-7288) targeted against the VP24 and NP of MARV-Musoke variant, respectively, was observed in 100% of NHP receiving 30 or 40 mg/kg, when treatments were initiated 30–60 min post-viral challenge. Similarly, 62.5% (5 out of 8) NHP infected with EBOV were protected by AVI-6002, a combination PMO*plus* therapeutic consisting of AVI-7537 and AVI-7539, targeting the EBOV VP24 and VP35 transcripts, respectively, when given at 40 mg/kg. It was subsequently shown that the combination therapeutic design for AVI-6002 and AVI-6003 was not essential to the protection of the NHP, and that only the oligonucleotides targeting VP24 and NP were required for protection against EBOV and MARV, respectively (Iversen et al., 2012). These PMO*plus* therapeutics have been under development by Sarepta Therapeutics funded by the US Department of Defense (US DoD), and have been tested in phase 1 clinical trials demonstrating their safety in healthy human volunteers between ages 18–50 (Iversen et al., 2012; Heald et al., 2014). Programs such as these are expensive to the US DoD due to the design of the antisense molecule and the extensive testing required under the US FDA Animal Rule, but great successes are being seen in these areas because the molecules appear safe and effective in animal model studies and phase 1 human clinical trials.

A very small handful of therapeutics such as ribavirin, interferons, recombinant human activated protein C (rhAPC) and recombinant nematode anticoagulant protein c2 (rNAPc2) have shown variable levels of efficacy against EBOV and MARV infections in animal models of infection (Huggins, 1989; Geisbert et al., 2003, 2007; Hensley et al., 2007; Smith et al., 2013). Interferons are commercially available and have been used in humans for treatment of other viral infections, but anti-filoviral efficacy in NHP models is uncertain and this treatment modality has a high risk of serious adverse events. These generally unproven, early-stage therapeutics are not under consideration for rapid development in the fight against the 2014 EBOV outbreak at this time.

### THERAPEUTIC VACCINES

Several anti-filovirus vaccine platforms, such as a replication-deficient recombinant adenovirus vaccine (Sullivan et al., 2003) which is soon to begin clinical trials in support of the West African EBOV outbreak; a live recombinant parainfluenza virus vaccine (Bukreyev et al., 2007); a live-attenuated, replication competent recombinant vesicular stomatitis virus (rVSV) vaccine against EBOV, SUDV, and MARV (Daddario-DiCaprio et al., 2006; Feldmann et al., 2007; Geisbert et al., 2008); a Venezuelan equine encephalitis virus RNA replicon vaccine [Drs John Dye and Gene Olinger, personal communication]; and Marburgvirus-like and Ebolavirus-like particles (Warfield et al., 2007; Warfield and Aman, 2011; Martins et al., 2013), and possibly even some others, are in development studies and are proving to be highly effective for preventing filovirus infections in non-human primates.

Of these, only the rVSV vaccine platform has also been reported to show post-exposure prophylactic efficacy in non-human primates, and post-exposure efficacy has been reported for this platform against MARV, EBOV, and SUDV in rhesus monkeys (Daddario-DiCaprio et al., 2006; Feldmann et al., 2007; Geisbert et al., 2008). Of note, while these vaccines are described as successful post-exposure protective measures, there are no reports of successful protection derived from these vaccines once clinical disease signs are apparent; these studies only tested the vaccines’ efficacy 30 min after parenteral virus exposure. In one additional study, the MARV construct was tested for its efficacy up to 48 h post-virus challenge, yet only 2 out of 6 animals survived (Geisbert et al., 2010a). For these filovirus vaccine constructs, the open reading frame encoding the filovirus transmembrane viral glycoproteins (GPs) was cloned into a VSV vector lacking the G gene (VSVΔG/X-GP, where X can be the GP for MARV, EBOV, or SUDV; Garbutt et al., 2004; Feldmann et al., 2007). For EBOV studies, eight rhesus monkeys were administered 2 × 10^7^ PFU of VSVΔG/EBOVGP, delivered intramuscularly to right and left caudal thigh muscles and right and left triceps, 20-30 min after intramuscular challenge with ∼1,000 PFU of EBOV (Feldmann et al., 2007). Two animals, receiving equivalent doses of control VSV vectors expressing either Marburgvirus GP or Lassa virus GP (VSVΔG/LASVGP), were included as experimental controls. While both control animals died on day 8 after infection after having developed characteristic signs of Ebola hemorrhagic fever 50% of the VSVΔG/EBOVGP-treated animals survived infection. A fifth VSVΔG/EBOVGP-treated animal succumbed on day 18 after infection, from an apparent bacterial infection of unknown origin. Three remaining VSVΔG/EBOVGP-treated animals succumbed on days 9 and 10 after infection. Day 6 viremia in animals that survived infection was reduced ∼2–4 log_10_ relative to viremia in animals that succumbed to infection.

The therapeutic potential of the rVSV vaccine platform has been similarly evaluated against SUDV (Geisbert et al., 2008). Five rhesus monkeys were infected with 1,000 PFU of SUDV, and 20–30 min afterward rVSV expressing the GPs of SUDV (VSVΔG/SUDVGP) was administered to four animals at four different i.m. sites at a dose of ∼2 × 10^7^ PFU, whereas the control animal received an equivalent dose of VSVΔG/LASVGP to control for possible non-specific effects due to the vector. All four VSVΔG/SUDVGP-treated monkeys survived the infection, and the control animal succumbed on day 17. SUDV burden in blood was reduced in all survivors relative to that of the control animal and remained undetected in two of these animals, and most of the animals were clinically appeared well throughout the study (Geisbert et al., 2008). The VSV platform is known to potently and quickly elicit a host innate immune response, followed by B- and T-cell driven specific immune responses, which are thought to be the mechanism of therapeutic action(s) for this vaccine, when used as a post-exposure therapeutic. Human clinical trials for the VSV platform began in the fall of 2014.

## CONCLUSION

Basic research to find filovirus therapeutics has been ongoing for 20 years, and now comes the most exciting time for this field of study, when some of the long-studied candidate therapeutics now have a chance at being tested under emergency use or in a clinical trial in support of the control of the Ebola outbreak in West Africa. Human safety is still a high priority when testing these novel classes of therapeutics, some of which are heretofore untested in humans under well-controlled clinical trial settings, but it is the opinion of the WHO and world virologists that the benefits of some protection against EBOV infection or death due to advanced stages of disease far outweigh the risks of administering an untested candidate therapeutic (Goodman, 2014; Sayburn, 2014). No matter what the outcomes of the studies, valuable data will be collected in support of diagnostic test development and the efficacies of candidate therapeutics when these new molecules and vaccines are tested under emergency use situations. With time and testing, the diagnostic tools will get more sensitive, and alert clinicians to filovirus infections earlier, and new therapeutics will be available for administration to help combat infections.

## Conflict of Interest Statement

The authors declare that the research was conducted in the absence of any commercial or financial relationships that could be construed as a potential conflict of interest. The Associate Editor, Fatah Kashanchi, declares that, despite being a co-author on a paper with the author Sina Bavari in the last 2 years, the review process was handled objectively and no conflict of interest exists.
